# Effectiveness of Ceftazidime-Avibactam in Gram-Negative Nosocomial Pneumonia: A Real-World Study in India

**DOI:** 10.7759/cureus.54443

**Published:** 2024-02-19

**Authors:** Neha Gupta, Sanjith Saseedharan, Yashesh Paliwal

**Affiliations:** 1 Internal Medicine and Infectious Diseases, Fortis Memorial Research Institute, Gurugram, IND; 2 Critical Care, SL Raheja Hospital - A Fortis Associate, Mumbai, IND; 3 Critical Care, Fortis Hospital, Kolkata, IND

**Keywords:** microbiological recovery, clinical cure, ventilator-associated pneumonia, nosocomial pneumonia, hospital-acquired pneumonia, ceftazidime-avibactam

## Abstract

Background and objective: The incidences of nosocomial pneumonia in intensive care units (ICUs) in India have been reported to range from 9% to 58% and are associated with a mortality rate of 30-70%. Ceftazidime-avibactam has activity against OXA-48-like carbapenem-resistant *Enterobacterales* (CRE) and has a safer adverse effect profile as compared to the nephrotoxic colistin. The current study aimed to assess the effectiveness and usage pattern of ceftazidime-avibactam in gram-negative hospital-acquired pneumonia (HAP) and ventilator-associated pneumonia (VAP) in real-world settings in India.

Methods: Electronic medical records of hospitalized patients in three prominent medical centers in India (Fortis Memorial Research Centre, Gurugram, S L Raheja Hospital, Mumbai, and Fortis Hospital, Anandapur, Kolkata) with nosocomial pneumonia and documented gram-negative *Klebsiella pneumoniae *(KP)*-*confirmed infection were collected. This study assessed the effectiveness, usage pattern of ceftazidime-avibactam, and clinical and microbiological cure rates.

Results: Among the 116 patients included, 78.45% (91/116) showed clinical cure. Microbiological cure was observed in nine out of 13 (69.23%) patients. In the subset analysis, a clinical cure rate of 84.85% (28/33) and microbiological recovery rate of 62.50% (5/8) were observed when ceftazidime-avibactam was initiated within 72 hours of diagnosis. Ceftazidime-avibactam was administered for a mean (±SD) duration of 7.79 ± 4.43 days, with improvement in signs and symptoms reported among 91.38% (106/116). Ceftazidime-avibactam showed a susceptibility of 56% (28/56) in the study.

Conclusion: The current study showed a better clinical and microbiological cure rate with a safer tolerability profile of ceftazidime-avibactam in carbapenem-resistant KP nosocomial pneumonia and VAP. This study has further demonstrated that ceftazidime-avibactam may be used as one of the viable treatment choices in carbapenem-resistant KP with favorable clinical outcomes.

## Introduction

Nosocomial pneumonia (NP) or hospital-acquired pneumonia (HAP) is one of the most common hospital-acquired infections, with high rates of mortality accounting for substantially greater healthcare costs [1-3]. Incidence rates of NP in intensive care units (ICUs) in India range from 9% to 58% and are associated with mortality rates of 30-70% [4]. Long hospitalization, immunosuppression, prolonged mechanical ventilation, and infections due to gram-negative bacteria with resistance to carbapenems and colistin contribute to the overall increased mortality of NP [5].

Gram-negative bacteria (*Pseudomonas aeruginosa*, *Escherichia coli*, *Klebsiella pneumoniae *(KP), *Enterobacter* spp., and *Acinetobacter* spp.), particularly *KP* and *P. aeruginosa*, have been found to be primarily responsible for the majority of NP than gram-positive bacteria [6-8].

A prospective study from southern India by Saroja et al. reported the incidence of HAP to be 12.5% among ICU patients [9]. Another prospective study by Buch et al. from western India found the incidence of HAP to be 9.42% [4,10]. According to Choudhuri et al., in a retrospective observational study from north India, 58.86% had an ICU-acquired nosocomial infection out of 153 ICU patients, with pneumonia (33%) as the most common infection [11].

India is a hotspot for carbapenem-resistant (CR) KP, with resistance reported ranging from 12% to an alarming 83% in Indian ICUs [9]. For a long time, polymyxins have been used as the backbone for the treatment of CRE HAP-ventilator-associated pneumonia (VAP). However, there has been an increasing concern regarding colistin resistance (up to 40% among CR KP) and the associated toxicity profile of colistin, resulting in a need for safer and more effective treatment options [12].

A novel βeta-lactam-βeta-lactamase inhibitor (BL-BLI), ceftazidime-avibactam is approved for the treatment of complicated urinary tract infections (cUTIs), complicated intrabdominal infections (cIAIs) and HAP-VAP, and secondary sepsis due to the above causes [13,14]. Avibactam, a non-BL-BLI, acts against class A (KPCs), class C, and some class D (OXA) β-lactamases and prevents ceftazidime from degradation, enhancing its action against *Enterobacterales* [15].

Among the CR KP, the prevalence of OXA-48 and OXA-48 like βeta-lactamases has been reported to be 28-50% in India [16,17,18]. Because of increasing concerns related to polymyxins, ceftazidime-avibactam is increasingly being used as one of the preferred options for the management of serious CRE infections in India. However, there are limited data available from real-world studies in HAP/VAP, and it is pertinent to assess ceftazidime-avibactam in real-world Indian clinical settings. Therefore, the current study aimed to assess the effectiveness and usage pattern of ceftazidime-avibactam in gram-negative HAP-VAP in the Indian real-world scenario.

## Materials and methods

We conducted a comprehensive retrospective observational study spanning three prominent medical centers in India (Fortis Memorial Research Centre, Gurugram, S L Raheja Hospital, Mumbai, and Fortis Hospital, Anandapur, Kolkata) with nosocomial pneumonia and documented gram-negative, covering the period from January 31, 2020, to January 31, 2021. The focus of the study was patients diagnosed with documented gram-negative carbapenem-resistant KP-associated nosocomial pneumonia, specifically those who underwent treatment with ceftazidime-avibactam for a minimum duration of 48 hours.

Inclusion and exclusion criteria

Patients with pre-existing gram-negative infections prior to hospital admission and those with bacterial isolates positive for metallo-β-lactamase (MBL) enzymes were systematically excluded. HAP was defined as pneumonia emerging 48 hours or more after hospital admission, excluding cases incubating at the time of admission. VAP was specifically delineated as pneumonia occurring beyond 48 to 72 hours post-tracheal intubation.

Data collection

A meticulous collection of study variables was undertaken from electronic medical records across three participating centers. This encompassed demographic information; clinical presentation; comorbidities; management protocols (including pharmacological interventions and additional measures, such as respiratory support); diagnostic details involving routine tests, culture, and susceptibility; recorded adverse events; and overall outcomes comprising clinical cure and microbiological cure, alongside morbidity and mortality at the time of discharge.

Outcomes

The primary focus of the study was to evaluate the clinical and microbiological efficacy of CAZ-AVI in treating gram-negative HAP-VAP patients. In addition, the study aimed to assess the usage patterns of CAZ-AVI in this specific patient cohort, serving as a secondary outcome.

Statistical analyses

Descriptive statistics, including proportions for categorical variables and mean values for continuous variables, were employed to present patient details, such as age, gender distribution, and culture isolates. The correlation between treatment specifics and patient profiles was scrutinized, with the significance level set at <0.05. Mortality and various clinical factors, including comorbidities, treatment modalities, and disease severity, were examined for the overall effects. Furthermore, the study delved into the outcome concerning the early initiation of CAZ-AVI.

Sample size determination

The sample size was meticulously calculated based on a presumed clinical cure rate of VAP at 60% (18), ensuring a precision level of <1% at a significance level of 5%. A statistical power analysis dictated a sample size of N = 100, maintaining a statistical power of 80% and an α error probability of 0.05.

The study adhered to ethical guidelines and received approval from the institutional review board or ethics committee at each participating center. Patient confidentiality and data privacy were strictly maintained throughout the study.

Recognized limitations, such as the retrospective nature of the study, were acknowledged. Efforts were made to mitigate biases through robust data collection and analysis.

## Results

In the present study, 116 patients were included, having a mean (±SD) age of 57.8 ± 18.69 years. Male patients outnumbered female patients by 74.14% (86/116) to 25.86% (30/116). Moreover, patients had a mean serum creatinine value of 1.62 ± 1.68 mg/dL. HAP with a median duration of five days of hospitalization was reported in 56% (65/116) patients, whereas VAP was diagnosed among 44% (51/116) patients with a median duration of hospitalization of 13 days (Table 1).

**Table 1:** Baseline characteristics and infection diagnosis details HAP: pneumonia that occurs within 48 hours or more after hospital admission without intubation. VAP: pneumonia that occurs after hospital admission for more than 48 to 72 hours post tracheal intubation. SD = standard deviation

Parameters	Pneumonia patients
Age (n=116)	
Mean ± SD	57.8 ± 18.69
Gender (n=116)	
Male	86 (74.14%)
Female	30 (25.86%)
Height (cm) (n=75)	
Mean ± SD	163.45 ± 8.97
Weight (kg) (n=82)	
Mean ± SD	62 ± 14.84
Serum creatinine (n=111)	
Mean ± SD	1.62 ± 1.68
Parameters	Pneumonia Patients
Types of Infection	
Hospital-acquired pneumonia (HAP) (n, %)	65 (56%)
Duration of HAP during hospitalization, median (IQR)	5 (7) days
Ventilator-associated pneumonia (VAP) (n, %)	51 (44%)
Duration VAP during hospitalization, median (IQR)	13 (11) days

The majority of patients had a history of hypertension (59.48%, 69/116), followed by diabetes (38.79%, 45/116). A history of chronic kidney disease, malignancy, thyroid, and chronic obstructive pulmonary disease (COPD) was reported in 24.14% (28/116), 22.41% (26/116), 12.93% (15/116), and 9.48% (11/116) patients (Table 2). COVID-19 was reported in 4.31% (5/116) of the patients. Cardiovascular (28.45%, 33/116), respiratory (47.41%, 55/116), and renal (13.79%, 16/116) complications were the most reported complications (Table 2).

**Table 2:** Medical history and complications* *One patient had multiple complications.

Parameters	N(%)
A. Medical history n (%)	
Hypertension	69 (59.48%)
Diabetes	45 (38.79%)
Chronic kidney disease	28 (24.14%)
Malignancy	26 (22.41%)
Thyroid disease	15 (12.93%)
Chronic obstructive pulmonary disease (COPD)	11 (9.48%)
Psychiatric condition	9 (7.76%)
Coronary heart disease	9 (7.76%)
Asthma	3 (2.59%)
Gastrointestinal disease	3 (2.59%)
COVID 19	5 (4.31%)
Others	66 (56.89%)
B. Complications	N(%)
Cardiovascular	33 (28.45%)
Myocardial infarction	8 (24.24%)
Hypotension	10 (41.67%)
Others	24 (72.73%)
Respiratory complications	55 (47.41%)
Acute respiratory distress syndrome	10 (8.62%)
Pleural effusion	10 (8.62%)
Pneumothorax	6 (5.17%)
Lung abscess	2 (1.72%)
Others	21 (18.10%)
Renal complications	16 (13.79%)
Acute kidney injury	10 (62.50%)
Renal failure	6 (37.50%)
Others	6 (37.50%)
Coagulation disorders	12 (7.23%)
Deep vein thrombosis	3 (25%)
Thrombocytopenia	7 (77.77%)
Others	9 (75%)
Gastrointestinal tract (GIT)	7 (6.03%)
Cerebrovascular	15 (12.93%)
Seizures	4 (26.67%)
Stroke	5 (33.33%)
Others	8 (53.33%)
Others	59 (50.86%)

Of all the sputum samples (n = 116), 51.72% (60/116) were found to be culture-positive on day 0. In the case of urine samples and blood samples, 20.69% (24/116) and 72.41% (84/116), respectively, were found to be culture-positive on day 0. KP was the most commonly detected bacteria in all the samples tested (100% in sputum (60/60), 100% in urine (24/24), and 100% in blood (84/84)), and among them, most were carbapenem-resistant (100% in sputum (60/60), 100% in urine (24/24), and 96.43% in blood (81/84)). The most common genotype observed was bLA-OXA-48:D (71.34%, 5/7) and was tested only in seven patients (Table 3). 

**Table 3:** Microbiological testing * Mixed infection, # *Klebsiella pneumoniae* identified, ^ XPERT-CARBA R (n = 6) and BioFire Pneumonia (n = 1) methods used to find the genotype, $ 2 (28.66%) carbapenem-resistant *Enterobacterales*. HAP: hospital-acquired pneumonia; VAP: ventilator-associated pneumonia

Parameters	Day 0 of VAP/HAP diagnosis	Repeat 1	Repeat 2
Sputum (n, %)			
Total (no. of cases)	116	13	1
Organism detected	60 (51.72%)	0 (0%)	0 (0%)
Organism not detected	56 (48.28%)	13 (100%)	1 (100%)
Bacteria/organism (no. of cases)	60	0	0
Klebsiella pneumoniae	60 (100%)	-	-
Acinetobacter*	1 (1.67%)	-	-
Carbapenem-resistant *K. pneumoniae*	60 (100%)	-	-
Urine (n, %)			
Total (no. of cases)	116	13	1
Organism detected	24 (20.69%)	2 (15.38%)	0 (0%)
Organism not detected	92 (79.31%)	11 (84.62%)	1 (100%)
Bacteria/organism (no. of cases)	24	2	0
Klebsiella pneumoniae	24 (100%)	2 (100%)	-
Carbapenem-resistant* K. pneumoniae*	24 (100%)	-	-
Blood (n, %)			
Total (no. of cases)	116	13	1
Organism detected	84 (72.41%)	1 (7.69%)	0 (0%)
Organism not detected	32 (27.59%)	11 (84.62%)	1(100%)
Report pending	-	1 (7.69%)	-
Bacteria/organism (no. of cases)	84	1	0
Klebsiella pneumoniae	84 (100%)	1 (100%)	-
Enterobacter cloacae*	1 (1.19%)	-	-
Pseudomonas aeruginosa*	1 (1.19%)	-	-
Carbapenem-resistant *K. pneumoniae*	81 (96.43%)	-	-
Others (n, %)			
Organism detected from other sources^#^	11 (9.48%)	0 (0%)	0 (0%)
ET culture	9 (81.81%)	-	-
Bronchoalveolar lavage	1 (9.09%)	-	-
Respiratory sample	1 (9.09%)	-	-
Genotype (n = 7^$^)^^^			
bLA-OXA-48: D	5 (71.34%)	-	

The susceptibilities of piperacillin-tazobactam, meropenem, imipenem, amikacin, and gentamicin were 14.12% (12/85), 17.74% (11/62), 13.48% (12/89), 22.68% (22/97), and 21.43% (18/84), respectively. Colistimethate sodium showed an intermediate susceptibility of 97.06% (99/102) and resistance of 2.94% (93/102) by the VITEK method (Table 4). The susceptibility of ceftazidime-avibactam was detected to be 56% (28/50) using the ETEST (Biomeriuex, Marcy-I'Etoile, France) method (Table 4).

**Table 4:** Antimicrobial resistance pattern S: susceptibility, I: intermediate, R: resistant, HAP: hospital-acquired pneumonia, VAP: ventilator-associated pneumonia

Parameters	Day 0 of VAP/HAP diagnosis			
Antibiotics	Total	S	I	R
Piperacillin-tazobactam	85	12 (14.12%)	0 (0%)	73 (85.88%)
Meropenem	62	11 (17.74%)	1 (1.61%)	50 (80.65%)
Imipenem	89	12 (13.48%)	9 (10.11%)	68 (76.4%)
Amikacin	97	22 (22.68%)	8 (8.25%)	67 (69.07%)
Gentamicin	84	18 (21.43%)	2 (2.38%)	64 (76.19%)
Ceftazidime-avibactam	50	28 (56%)	0 (0%)	22 (44%)
Colistimethate sodium	102	-	99 (97.06%)	3 (2.94%)

The clinical cure and microbiological recovery rates were 78.45% (91/116) and 69.23% (9/13) on a median seven-day assessment, respectively. Of 33 patients, to whom ceftazidime avibactam was initiated within 72 hours of diagnosis, 84.85% of the patients (28/33) showed clinical recovery and 62.50% (5/8) showed microbiological recovery on a median five-day assessment (Table 5).

**Table 5:** Recovery rate in patients diagnosed with HAP/VAP *Resolution of clinical symptoms recorded at the time of HAP/VAP diagnosis. # Clearance of infections/gram-negative bacteria. & Resolution of pneumonia findings in radiological tests. ~ Median (IQR) days of assessment. HAP: hospital-acquired pneumonia, VAP: ventilator-associated pneumonia CAZ/AVI - Ceftazidime avibactam Clinical cure was considered when there was a resolution of all new signs and symptoms. Microbiological cure was considered when there was a resolution of all pathogens.

Parameter	Outcome	
	Clinical cure*	Microbiological cure^#^
Total number of patients diagnosed with HAP/VAP (n=116)		
Number of patients recovered	91/116	9/13
Effectiveness	78.45%	69.23 %
Median (IQR) days^~^	7 (19)	7 (7.5)
Range	1-184	1-29
CAZ-AVI initiated within 72 hours of diagnosis of HAP/VAP (n=33)		
Number of patients recovered	28/33	5/8
Effectiveness	84.85%	62.50%
Median (IQR) Days^~^	5 (6)	5 (12)
Range	1-158	1-29

Ceftazidime-avibactam intravenously was initiated as empiric therapy in 50% of patients (58/116) and after susceptibility in 50% of patients (58/116). The most prescribed dose was 2.5 mg three times a day (TDS) (75.86%, 88/116), with a mean duration of treatment of 7.79 days ± 4.43 days; 106/116 patients (91.38%) showed an improvement in signs and symptoms on a median of seven days with the treatment (Table 6).

**Table 6:** Treatment details *Other doses of the drug: 0.5 gm (1), 0.75 gm (2), 0.90 gm (2), 0.94 gm (1), 0.95 gm (1), 1 gm (6), 2.2 gm (2) # Patients requiring mechanical ventilation during the treatment period. CPAP: continuous positive airway pressure, BPAP: bilevel positive airway pressure, OD: once daily, BD: two times a day, TDS: three times a day, VAV: variable air volume, AC: assist-control, VCV: volume-controlled ventilation, PCV: pressure-controlled ventilation, PEEP: positive end-expiratory pressure, CPR: cardiopulmonary resuscitation

Parameters	N(%)
Ceftazidime-avibactam	
Initiated as empiric therapy (n=116)	N(%)
Yes	58 (50%)
No	58 (50%)
Initiated after culture susceptibility (n=116)	
Yes	58 (50%)
No	58 (50%)
Dose (n=116)	
Frequency of 2.5 gm (n=87)	87 (75.00%)
OD	3 (3.45%)
BD	7 (8.05%)
TDS	77 (88.51%)
Frequency of 2.25 gm (n=6)	6 (5.17%)
TDS	6 (100%)
Frequency of 1.25 gm (n=13)	13 (11.21%)
BD	6 (46.15%)
TDS	7 (53.85%)
Frequency of 1.5 gm (n=5)	5 (4.31%)
BD	1 (20%)
TDS	4 (80%)
Others*	
Duration of treatment prescribed (days) (Mean ± SD) (n=116)	7.79 ± 4.43
Route of administration (n=116)	
Parenteral (IV)	116 (100%)
Failure of improvement in signs and symptoms (n=116)	
Yes	10 (8.62%)
No	106 (91.38%)
Oxygenation (n=116)	
Yes	30 (25.86%)
Rate of flow of oxygen (mean’s)	8.38 ± 5.56 (16)
Mechanical ventilation (n=116)	
Yes^#^	34 (29.31%)
CPAP	9 (26.47%)
BPAP	12 (35.29%)
Others	13 (11.20%)
-VAV, AC mode	6 (46.15%)
-VCV	4 (30.77%)
-T piece, VCV	1 (7.69%)
-VC, AC mode	1 (7.69%)
-PCV, AC, PEEP-8, 9, 11	1 (7.69%)
Dialysis proposed in patients (n=12)	
Patients underwent dialysis	4 (25%)
CPR (n=116)	
Patients requiring CPR	9 (7.76%)

A TDS dose of 2.5 mg was the most prescribed dose among stage 1 (GFR >90 mL/min/1.73 m^2^), stage 2 (GFR: 60-89 mL/min/1.73 m^2^), stage 3a (GFR: 45-59 mL/min/1.73 m^2^), and stage 3b (GFR: 30-44 mL/min/1.73 m^2^) patients at admission and at day 0 (Table 7).

**Table 7:** Concomitant antibacterial medications in the management of HAP/VAP HAP: hospital-acquired pneumonia, VAP: ventilator-associated pneumonia

Medicines	N(%)	Mean duration (days)
Antibacterial drugs (n=116)		
Meropenem	78 (67.24%)	9.9
Teicoplanin	71 (61.21%)	8.6
Polymyxin B	70 (60.34%)	7.8
Aztreonam	57 (49.14%)	7.1
Colistimethate sodium	36 (31.03%)	7.9
Doxycycline	26 (22.41%)	7.8
Metronidazole	25 (21.55%)	7.8
Linezolid	22 (18.97%)	7.6
Piperacillin + tazobactam	20 (17.24%)	6.1
Minocycline	14 (12.07%)	6.6
Cefoperazone + sulbactam	13 (11.21%)	5.9
Sulfamethoxazole + trimethoprim	13 (11.21%)	6.9
Ceftriaxone	12 (10.34%)	3.8
Levofloxacin	12 (10.34%)	10.6
Tigecycline	12 (10.34%)	7.9
Amikacin	11 (9.48%)	5.2
Fosfomycin	10 (8.62%)	5.7
Clarithromycin	9 (7.76%)	5.1
Clindamycin	9 (7.76%)	5.3
Vancomycin	9 (7.76%)	5.8
Cyclosporine	6 (5.17%)	11.0
Cefuroxime	5 (4.31%)	6.8
Ciprofloxacin	5 (4.31%)	9.8
Gentamycin	5 (4.31%)	11.0
Rifaximin	5 (4.31%)	10.0
Azithromycin	4 (3.45%)	8.3
Faropenem	4 (3.45%)	3.0
Imipenem	4 (3.45%)	9.0
Cefepime + tazobactam	3 (2.59%)	6.7
Cefixime	3 (2.59%)	10.0
Nitazoxanide	3 (2.59%)	3.3
Ofloxacin	3 (2.59%)	7.7
Amphortecin B	2 (1.72%)	2.5
Ceftriaxone + disodium edetate + sulbactam	2 (1.72%)	5.0
Cilastatin	2 (1.72%)	7.5
Dorepenem	2 (1.72%)	18.5
Ertapenem	2 (1.72%)	2.5
Menocyclin	2 (1.72%)	3.5
Moxifloxacin	2 (1.72%)	5.0
Nitrofurantoin	2 (1.72%)	10.5
Tobramycin	2 (1.72%)	6.0
Acetycysteine	1 (0.86%)	3.0
Ambisome	1 (0.86%)	3.0
Artesunate	1 (0.86%)	5.0
Cefadroxil	1 (0.86%)	4.0
Chromobact	1 (0.86%)	5.0
Colostin	1 (0.86%)	4.0
Ethambutol	1 (0.86%)	20.0
Human normal immunoglobulin	1 (0.86%)	1.0
Isepamicin	1 (0.86%)	5.0
Levetiracetam	1 (0.86%)	14.0
Monobactam	1 (0.86%)	4.0
Monurol sachet	1 (0.86%)	7.0
Mupirocin	1 (0.86%)	3.0
Norfloxacin + lactobacillus	1 (0.86%)	4.0
Ofloxacin + ornidazole	1 (0.86%)	-
Posaconazole	1 (0.86%)	6
Rifampicin + isoniazid	1 (0.86%)	22
Sulbactam	1 (0.86%)	1
Tazobactam	1 (0.86%)	4

The most common concomitant medications were antibacterial drugs, including meropenem (78 (67.24%)), teicoplanin (71 (61.21%)), and polymyxin B (70 (60.34%)) (Table 8).

**Table 8:** Ceftazidime-avibactam treatment details based on the glomerular filtration rate GFR: glomerular filtration rate, OD: once daily, BD: twice daily, TID: thrice daily

GFR	Number of patients	Dose (mg)	Frequency			Duration (days)	
			OD	BD	TID	Mean	Min-Max
At admission (n=19)							
Stage	3 (15.79%)	0.95 mg (2) and 2.5 mg (1)	-	-	3	10	2-21
Stage 3a	1 (5.26%)	2.5 mg	-	-	1	12	12-12
Stage 3b	15 (78.95%)	2.5 mg (11), 1.25 mg (2), and 1 mg (2)	-	1	14	7.73	1-17
Day 0 (n=16)							
Stage 1	4 (25%)	2.5 mg (3) and 2.25 mg (1)	-	-	4	10.5	7-15
Stage 2	2 (12.5%)	2.5 mg (2)	-	-	2	5.5	5-6
Stage 3a	5 (31.25%)	2.5 mg (3), 1.25 mg (1), and 1 mg (1)	-	1	4	7.2	3-11
Stage 3b	5 (31.25%)	2.5 mg (1), 1 mg (2), 0.5 mg (1), and 0.95 mg (1)	1	-	4	12.4	7-21

The median duration of hospitalization was 21 days. All the hospitalized patients (100%, 116/116) were admitted to the ICU for a median duration of 16 days. In-hospital complications were observed in 58.62% (68/116) patients. Approximately 33 (28.45%) patients died during hospital treatment, and one patient died post readmission at the time of discharge. The overall mortality seen in the patients was 29.31% (34/116) (Table 9).

**Table 9:** Hospitalization details # Overall patients requiring mechanical ventilator

Parameters	N(%)
Duration of hospitalization (days) (n=116)	
Median (IQR)	21 (17.75)
ICU admission (n=116)	
Yes	116 (100%)
Duration of ICU stay (days), median (IQR)	16 (15.75), 19.09 ± 13.71
In-hospital complications (n=116)	
Yes	68 (58.62%)
Mechanical ventilator (n=116)	
Mechanical ventilator^# ^	53 (45.69%)
Duration of mechanical ventilation (days), median (IQR)	11 (20)
Overall mortality (n=116)	
Dead	34 (29.31%)

## Discussion

A considerable proportion of hospitalized patients often develop nosocomial bacterial infections like HAP and VAP, leading to clinical complications. In recent studies, these nosocomial infections were found to be significantly associated with worse outcomes and deaths despite the use of antimicrobial agents [14,19]. Several clinical trials have proven the efficacy of CAZ-AVI in the treatment of NP, but the data from the real world still remain scarce. The current study aimed to establish the effectiveness and safety of CAZ-AVI in the treatment of NP in an Indian real-world setting.

In this study, 116 patients were enrolled, showcasing a male predominance of 74.14% (86/116). HAP with a median duration of five days of hospitalization was reported in 56% (65/116) patients, whereas VAP was diagnosed in 44% (51/116) patients with a median duration of 13 days of hospitalization. Bhadade et al., in a prospective observational cohort study, reported 24% of patients with HAP and 76% of patients with VAP [4]. Soriano et al. reported that out of 516 patients treated by CAZ-AVI for at least 72 hours, the main indications were HAP/VAP (22.1%) [19]. Several Indian studies found a predominance of the male population in HAP/VAP patients, similar to our study [20,21].

Pneumonia caused by gram-negative, encapsulated KP is the most prevalent cause of HAP and responsible for approximately 11.8% of all HAP globally [22,23]. In a subset analysis from REPROVE by Sathe et al., *P. aeruginosa* was the most commonly isolated pathogen (53.8%), followed by KP (23.1%) [14]. Nagvekar et al. and Bhadade et al. found KP as the predominant pathogen causing VAP in Indian patients [4,24]. Similar results were also reported in various Indian studies where enterobacterales (like KP) and *P. aeruginosa* were common pathogens to be isolated [11,25,26]. Thus, KP, especially carbapenem-resistant strains, contributes significantly to HAP and VAP, which has implications for antibiotic resistance and calls for effective surveillance and infection control strategies.

In this study, CAZ-AVI showed susceptibility (56.0%, 28/50) on day 0 for carbapenem-resistant isolates. In Rathish et al.'s study, 79% of patients showed sensitivity of CRE isolates to CAZ-AVI [27]. Bakthavatchalam et al. also reported the overall susceptibility to CAZ-AVI as 72% among KP isolates, and further study reported that 51% of carbapenem-resistant KP were susceptible to CAZ-AVI [28].

In the REPROVE trial by Torres et al., CAZ-AVI was well tolerated in patients with HAP and VAP [29]. Similar results were also observed in our study, where 92.24% (107/116) patients continued with the prescribed dose and duration of CAZ-AVI, demonstrating good tolerability of the combination, which is consistent with established evidence on the safety profile of CAZ-AVI [30,31]. The CAVICOR study reported that in patients treated with CAZ-AVI, the 21-day clinical response was more frequent than in those treated with the best available therapy (BAT) (169/189, 89.4% vs. 119/150, 79.3%; P = 0.01). Furthermore, the patients with pneumonia treated with the CAZ-AVI group had a higher rate of 21-day clinical response than patients treated with BAT (21/23, 91.3% vs. 9/16, 56.2%; P = 0.01) [32]. Soriano et al.'s study reported 68.5% treatment success of CAZ-AVI in a combination therapy regimen. In addition, the study showed that 88.5% of all isolates and 92.4% of multidrug-resistant isolates of KP were resistant to carbapenems [19]. Jorgensen et al.'s study reported that CRE was isolated from 57.6% of culture specimens; among them, 63.2% were KP [33]. Similar clinical cure rates were reported in the CAZ-AVI combination therapy in a systematic review and meta-analysis [34,35].

In this study, the clinical resolution of HAP/VAP with CAZ-AVI was observed in 91/116 patients, suggesting an overall effectiveness rate of 78.45%. There was a trend seen in the effectiveness when the drug was initiated within 72 hours of diagnosis (84.85%, 28/33). Satlin et al. reported that patients whose blood cultures underwent KP carbapenemase gene (blaKPC) PCR testing had a shorter time until receipt of active therapy (median: 24 vs. 50 hours; P = 0.009) compared with other patients and decreased 14-day (16% vs. 37%; P = 0.007) and 30-day (24% vs. 47%; P = 0.007) mortality. Patients with CRE had also shown a delayed response to appropriate treatment and a high mortality rate, suggesting the need for rapid molecular diagnostics for carbapenemases to improve outcomes [36]. Previous studies have also reported that the early use of CAZ-AVI (within 48 hours of infection onset) is associated with improved clinical cure rates [33,37]. Jorgensen et al. showed that the clinical success rate was 33.3% and the failure rate was 18.6% when the treatment was initiated within 48 hours. Moreover, the failure rates increased as the time period for initiation of the drug increased [33].

Luna et al. reported that in some ICUs, organisms sensitive to colistin alone are responsible for >20% of gram-negative VAP [38]. Various studies have also reported that inhaled colistin reduces the duration of mechanical ventilation [39,40]. Colistin as an antibiotic has various limitations due to its narrow therapeutic index, challenges with dosage optimization, poor lung penetration, and nephrotoxicity [41]. Thus, CAZ-AVI can be a preferred alternative to colistin in the treatment of such infections from an antimicrobial stewardship perspective [14]. Wang et al., in a study of 30 patients on a CAZ-AVI combination, reported that 66.6% received target treatment, 26.7% received empirical treatment, and 6.7% received treatment with no indication [13]. In the present study, 50% (58,/116) patients received CAZ-AVI before, and 50% (58/116) patients received CAZ-AVI after susceptibility testing.

Wang et al.'s study recommended a dose as per drug instructions, which was administered to 50% of patients, at a median treatment duration of 10 days (range: two to 74 days) [13]. In the current study, the patients received CAZ-AVI via the parenteral route (100%) at a dose of 2.5 gm (75.86%, 88/116) for a mean (±SD) duration of treatment of 7.79 ± 4.43 days. An improvement in signs and symptoms with the treatment was reported in 91.38% (106/116) patients over a median of seven days. In this study, 92.24% (107/116) of the patients were continued CAZ-AVI with the prescribed dose and duration, demonstrating the good efficacy and tolerability of CAZ-AVI.

Merdjan et al. reported a decrease in the total plasma clearance with increasing severity of renal impairment in patients on avibactam, where avibactam was considerably removed via hemodialysis [42]. Sathe et al. reported that in the Indian subset, only 2.6% of patients had moderate to severe renal impairment, and a dose adjustment is recommended in patients with an estimated creatinine clearance <50 mL/min [14]. Nagvekar et al. also reported that 64.91% of patients require renal dose adjustment [24]. Another study by Rathish et al. reported that 25% (n = 26) of patients with impaired renal functions required renal dose modifications. However, the study showed no association between renal dose modification and mortality and was statistically not significant (P = 0.803) [27]. In this study, a TDS dose of 2.5 mg (CAZ-AVI) was the most prescribed dose in renal-impaired patients. However, it would be important to individualize the dosage modifications of ceftazidime-avibactam as per the prescribing information in renal-impaired patients.

In the current study, the most common antibacterial drugs are meropenem, teicoplanin, polymyxin B, and aztreonam. Similar findings were reported by Nagvekar et al., where the most prescribed drugs were aztreonam, polymyxin, tigecycline, and fosfomycin [24]. Rathish et al. also reported the addition of antibiotics with CAZ-AVI in 33% (n = 34) patients, which included tigecycline, minocycline, polymyxin, meropenem, levofloxacin, and aztreonam [27]. In addition, Soriano et al. found meropenem, piperacillin-tazobactam, and vancomycin as the most frequently used antibiotics [19]. 

We acknowledge the potential limitations of this study. These include the retrospective nature of data collection, which may be subjected to inherent biases and incomplete records. As an observational study, establishing causation between the use of ceftazidime-avibactam and clinical/microbiological outcomes is challenging. Despite statistical adjustments, the presence of unmeasured confounding variables could influence the observed associations between the use of ceftazidime-avibactam and outcomes. The study period covers a dynamic time frame (January 2020 to January 2021) marked by the COVID-19 pandemic. Changes in treatment protocols, healthcare practices, or patient management during this period could introduce variability and affect the study outcomes. We acknowledge the limitations of any study to provide a complete and transparent understanding of its scope and potential constraints.

Patients with HAP and VAP admitted to intensive care often require mechanical ventilation. Studies have found VAP to be associated with an overall increased stay in the ICU and hospital, in addition to the increased time under mechanical ventilation [1]. HAP and VAP are also associated with an increased risk of death, with a mortality rate, for HAP ranging from 38% to 70% [43]. In this study, 34/116 (29.31%) patients died due to various complications (multiple organ dysfunction, septic shock, respiratory, renal, and cardiovascular disorders). Nagvekar et al. reported an in-hospital death rate of 21% with the use of CAZ-AVI for the treatment of infections due to CRE in acutely ill patients [24]. Rathish et al. demonstrated overall mortality of 27%, similar to the findings reported by King et al. (32% mortality) and Shields et al. (24% mortality) when treated with CAZ-AVI [27,44,45]. Feng et. al. found that the risk factors for death in HAP patients included age >70 years, ICU admission, blood lymphocyte count, multidrug-resistant gram-negative bacteria (MDR-GNB) infection, and blood urea nitrogen (BUN) level [46]. Another study found the mortality rate in HAP patients to increase with increasing severity classification [47]. Thus, variables such as type of infection, the severity of illness at the time of diagnosis, antimicrobial susceptibility, renal impaired condition, and underlying comorbid conditions appear to play an important role in determining the prognosis of these patients.

## Conclusions

The current study provides compelling evidence for the effectiveness and tolerability of ceftazidime-avibactam in the treatment of carbapenem-resistant infections. This study has further demonstrated that ceftazidime-avibactam may be used as one of the preferred choices in carbapenem-resistant infections with favorable clinical outcomes. Integrating rapid diagnostic tools into clinical practice along with precise antibiotic selection can significantly improve the treatment outcomes in HAP/VAP cases. These findings support the utilization of ceftazidime-avibactam and emphasize the significance of incorporating rapid diagnostics into routine clinical management to optimize patient care.
